# Supplementary material for: Multiomic analyses uncover immunological signatures in acute and chronic coronary syndromes
Source: Nat Med. 2024 May 21;30(6):1696–710. doi: 10.1038/s41591-024-02953-4 (PMC11186793; doi:10.1038/s41591-024-02953-4)
Supplement: Supplementary file 1 — Supplementary Figs. 1–14. [file 41591_2024_2953_MOESM1_ESM.pdf]

nature medicine

Supplementary information https://doi.org/10.1038/s41591-024-02953-4

Multiomic analyses uncover immunological signatures in acute and chronic coronary syndromes

In the format provided by the authors and unedited

Multiomic analyses uncover immunological signatures in acute and chronic coronary syndromes

Kami Pekayvaz[1,2,*,#], Corinna Losert[3,4*], Viktoria Knottenberg[1*], Christoph Gold[1,2], Irene V. van Blokland[5,6], Roy Oelen[6], Hilde E. Groot[5], Jan Walter Benjamins[5], Sophia Brambs[1], Rainer Kaiser[1,2], Adrian Gottschlich[7,8], Gordon Victor Hoffmann[8], Luke Eivers[1], Alejandro Martinez-Navarro[1], Nils Bruns[1], Susanne Stiller[1], Sezer Akgöl[1], Keyang Yue[1], Vivien Polewka[1], Raphael Escaig[1], Markus Joppich[9], Aleksandar Janjic[10], Oliver Popp[11], Sebastian Kobold[8,12,13], Tobias Petzold[1,2,14,15,16], Ralf Zimmer[9], Wolfgang Enard[10], Kathrin Saar[11,15], Philipp Mertins[11], Norbert Huebner[11,15,16], Pim van der Harst[17], Lude H. Franke[6], Monique G. P. van der Wijst[6], Steffen Massberg[1,2], Matthias Heinig[2,3,4,#,*], Leo Nicolai[1,2,#,*] and Konstantin Stark[1,2,#,*]

[1]Medizinische Klinik und Poliklinik I, LMU University Hospital, Munich, Germany

[2]DZHK (German Centre for Cardiovascular Research), Partner Site Munich Heart Alliance, Germany

[3]Institute of Computational Biology, German Research Center for Environmental Health, Helmholtz Zentrum München, Neuherberg, Germany

[4]Department of Computer Science, TUM School of Computation, Information and Technology, Technical University of Munich, Garching, Germany

[5]Department of Cardiology, University of Groningen, University Medical Center Groningen, Groningen, The Netherlands

[6]Department of Genetics, University of Groningen, University Medical Center Groningen, Groningen, The Netherlands

[7]Department of Medicine III, LMU University Hospital, Munich, Germany

[8]Division of Clinical Pharmacology, LMU University Hospital, Member of the German Center for Lung Research (DZL), Munich, Germany

[9]Department of Informatics, Ludwig-Maximilians University, Munich, Germany

[10]Anthropology and Human Genomics, Faculty of Biology, Ludwig-Maximilian University, Munich, Germany

[11]Max Delbrück Center for Molecular Medicine in the Helmholtz Association (MDC), Berlin, Germany

[12]German Cancer Consortium (DKTK), a partnership between DKFZ and LMU University Hospital, Partner Site Munich, Germany

[13]Einheit für Klinische Pharmakologie (EKLiP), Helmholtz Zentrum München - German Research Center for Environmental Health Neuherberg, Germany

[14]Department of Cardiology, Angiology and Intensive Care Medicine, Deutsches Herzzentrum der Charité (DHZC), Campus Benjamin Franklin

[15]German Center for Cardiovascular Research (DZHK), Partner Site Berlin, Germany

[16]Charite-Universitätsmedizin Berlin, Berlin, Germany

[17]Department of Cardiology, University Medical Center Utrecht, Utrecht, The Netherlands

[#]Correspondence to: kami.pekayvaz@med.uni-muenchen.de, matthias.heinig@helmholtz-muenchen.de, leo.nicolai@med.uni-muenchen.de and konstantin.stark@med.uni-muenchen.de

*These authors contributed equally.

This document contains the following files:

Supplementary Figures

Supplementary Figures 1-14

Supplementary Figures

Suppl. Fig. 1

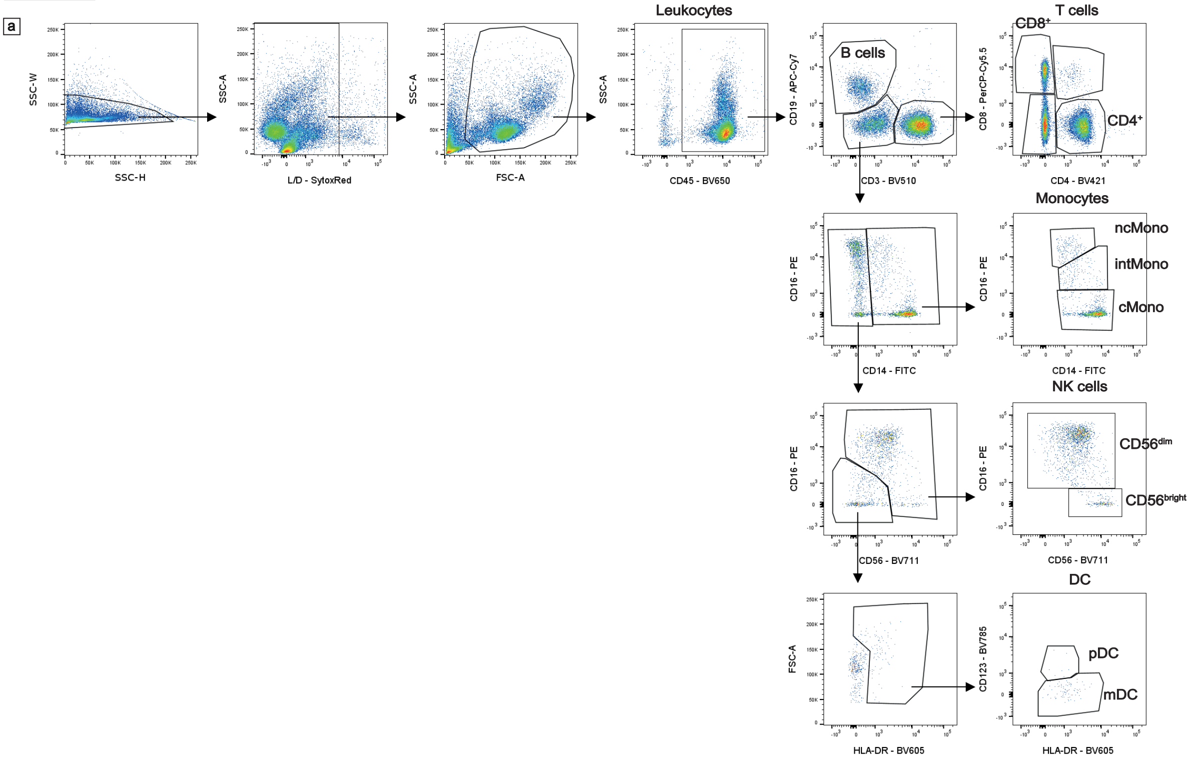

Suppl. Fig. 1

(a) Gating strategy used for flow-cytometric quantification of PBMCs in blood (Leukocytes, B cells, T cells ($CD8^+$ and $CD4^+$), Monocytes (non-classical monocytes (ncMono), intermediate monocytes (intMono), classical monocytes (cMono)), NK cells ($CD56^{dim}$ and $CD56^{bright}$), Dendritic cells (DC) (plasmacytoid dendritic cells (pDC), myeloid dendritic cells (mDC)).

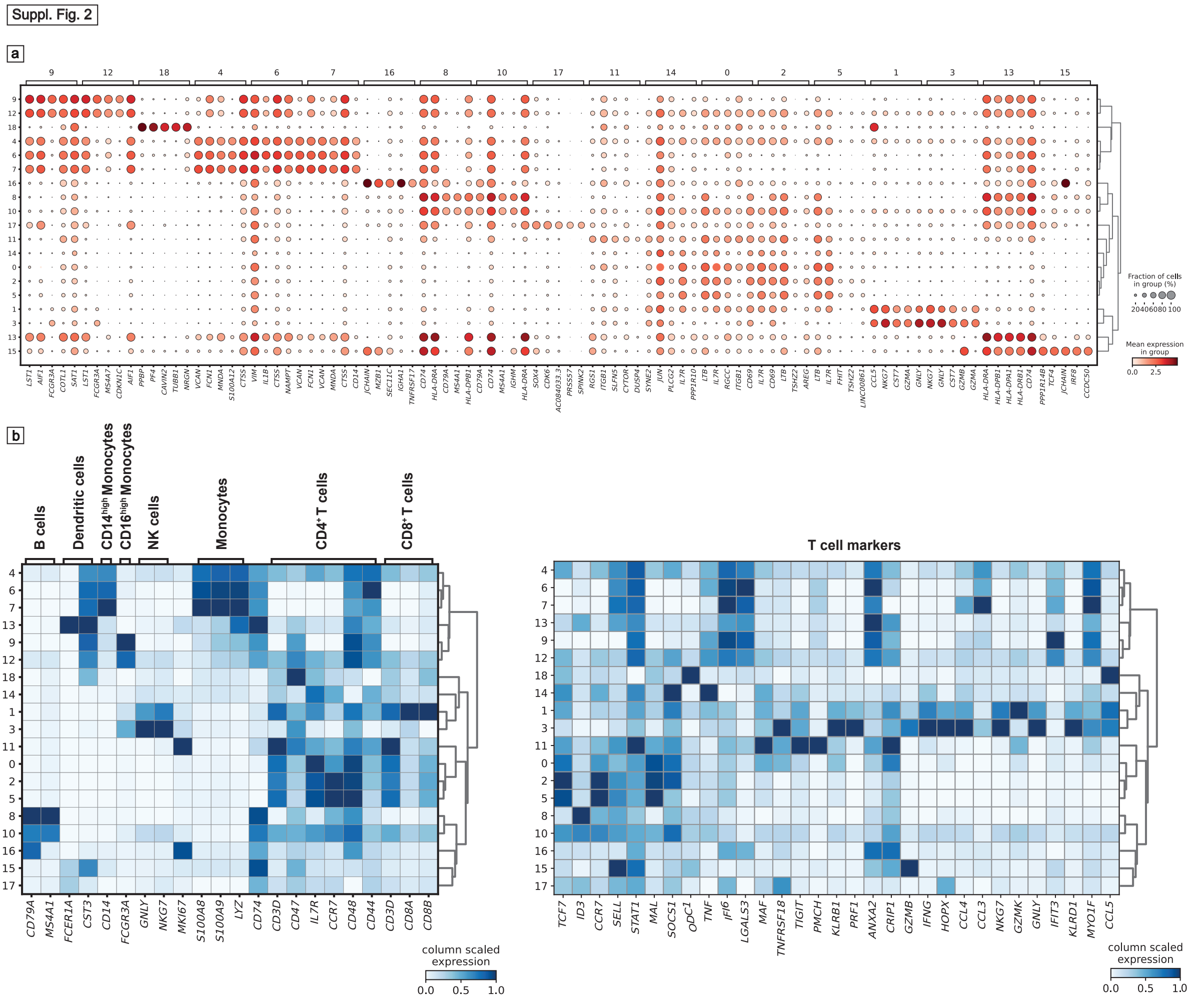

Suppl. Fig. 2

(a) Normalized and log-transformed mean expression of differentially expressed genes (bottom x-axis) shown across all clusters (y-axis). Differentially expressed genes calculated using a Wilcoxon rank sum test with Benjamini-Hochberg correction contrasting expression of one cluster (top x-axis) to expression of the other clusters. **(b)** Scanorama transformed mean expression of PBMC (left) and T cell marker (right) genes per cluster (y-axis) and annotated cell-types (top x-axis).

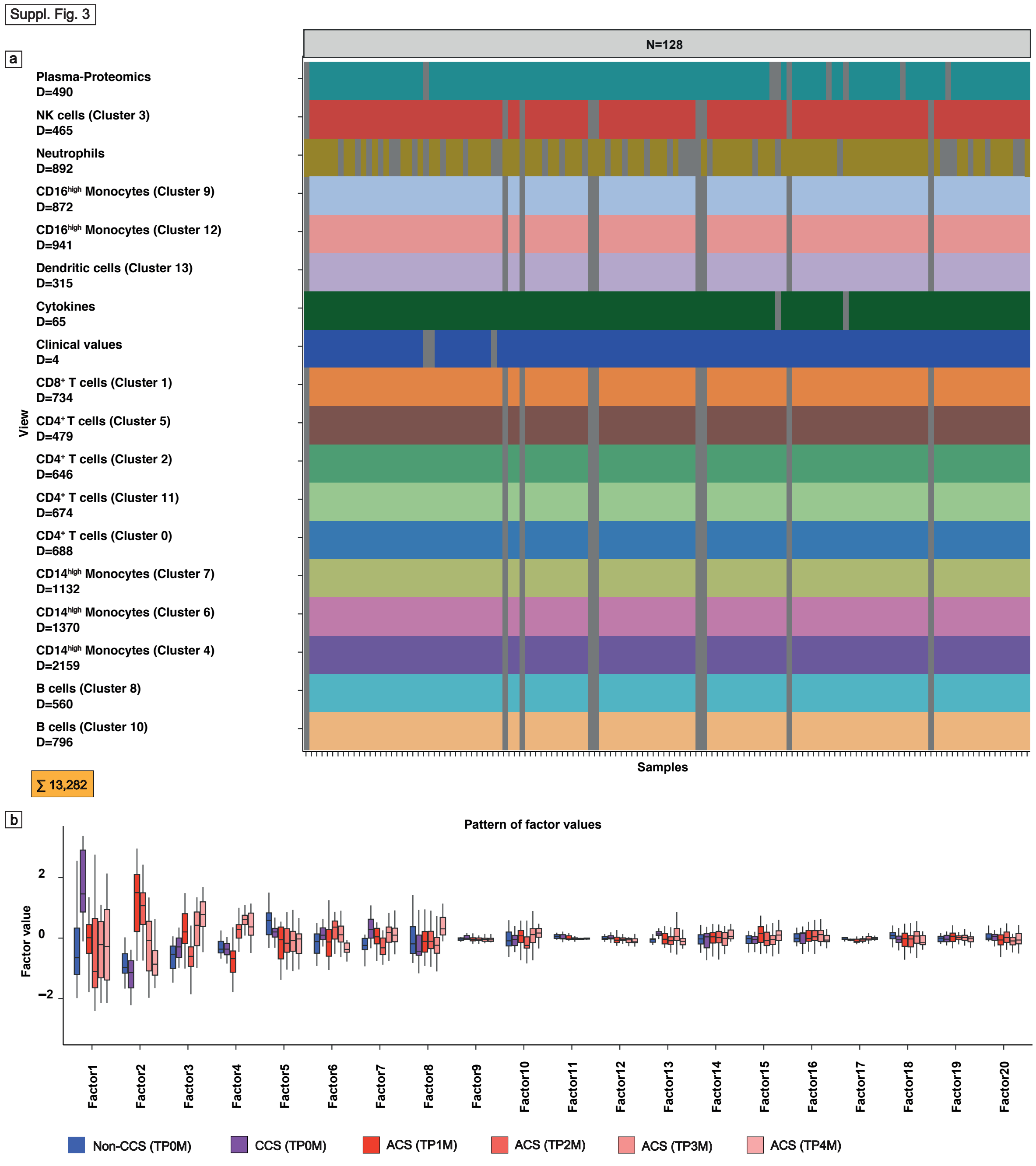

Suppl. Fig. 3

(a) Overview data input MOFA (exploratory Munich cohort): amount of features for each input data dimension (D), amount of samples (N, defined as combinations of subjects and timepoints TP1M/TP2M/TP3M/TP4M) and missing data highlighted by grey colors (specifying missing data for a specific dimension and sample). **(b)** Factor values of all samples (n=128) in longitudinal comparison for all 20 MOFA factors estimated based on input views visualized in

(a) (exploratory Munich cohort). Data are shown as a Box-Whiskers plot (box: median, 25th to 75th percentile; whiskers: hinge to largest/ smallest value, no further than 1.5*IQR from hinge).

Suppl. Fig. 4

a

Cell type Azimuth × Cluster cell type Scanorama (n)

Cell type Azimuth	CD4⁺ T cells (Cluster 0)	CD8⁺ T cells (Cluster 1)	B cells (Cluster 10)	T_{regs} cells (Cluster 11)	$CD16^{high}$ Monocytes (Cluster 12)	Dendritic cells (Cluster 13)	Other cells (Cluster 14)	CD4⁺ T cells (Cluster 2)	NK cells (Cluster 3)	$CD14^{high}$ Monocytes (Cluster 4)	CD4⁺ T cells (Cluster 5)	$CD14^{high}$ Monocytes (Cluster 6)	$CD14^{high}$ Monocytes (Cluster 7)	B cells (Cluster 8)	$CD16^{high}$ Monocytes (Cluster 9)	Total
Total	24806	19754	2897	2769	1934	1365	1345	19071	15670	14625	12514	11202	7836	7399	3114	
Treg	437	34	24	785	1		84	701		18	275			1		2362
NK		1379	53	1		3	41		14185	35						15699
MAIT	32	1085	11		4		8	1	15	20	1					1177
gdT	5	1652	7	7	2		8	30	176	9	5					1901
cDC2						1171				80		58	1			1314
CD8 TEM	87	9476	118	71	19		174	21	502	126	9					10611
CD8 Naive	40	5	8	13	2		8	1444		9	1083					2612
CD4 TEM	2638	2318	62	101	13		143	826	4	56	131		1	1		6296
CD4 TCM	21149	476	286	1571	90	1	626	14323	2	363	6201		2	1		45102
CD4 Naive	36	15	17	2	2		133	1524		9	4752			2		6495
CD4 CTL		2262	19	5	2		6		43	9						2346
CD16 Mono					1557					54		123	23		2920	4680
CD14 Mono		1			129	89	19	1		13466		11012	7789		156	32727
B naive			597		13		2			11				2935		3558
B memory			744		17	1	2			49				1175		2010
B intermediate			889		28	2	5			64				3270		4261

Colour scale (n): 10000, 20000, 30000, 40000

Cluster cell type Scanorama

b

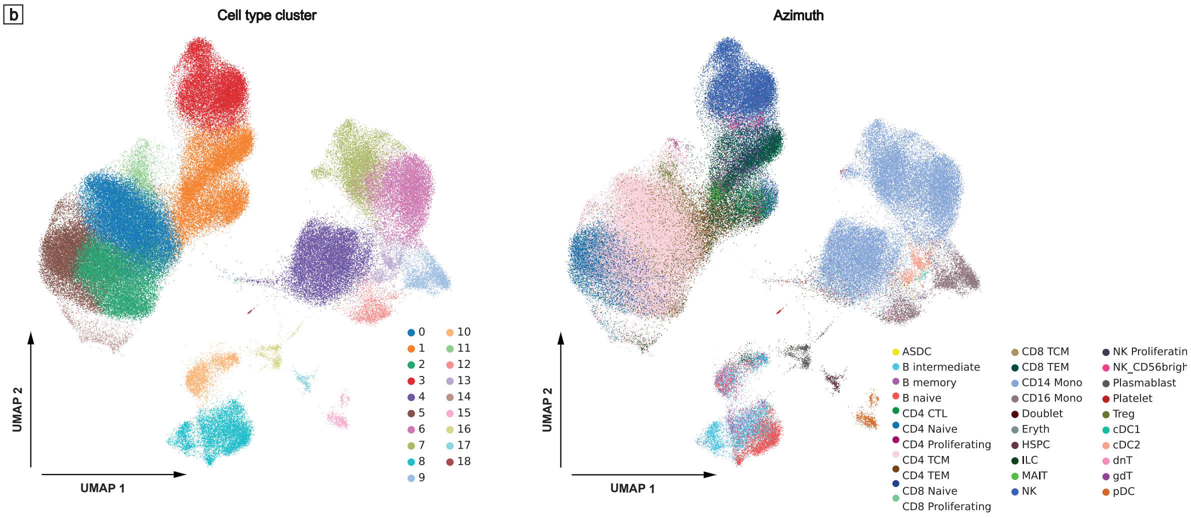

Suppl. Fig. 4

(a) Comparison of the cell-type annotation strategies on Munich data: x-axis showing clusters and assigned cell-types based on marker genes approach; y-axis showing automated cell-type annotations using the Groningen Azimuth annotation pipeline. **(b)** Comparison of the cell-type annotation strategies on Munich data as umap: left umap showing cell-type clusters that have been annotated based on marker genes approach; right umap showing automated cell-type annotations using the Groningen Azimuth annotation pipeline on the same umap.

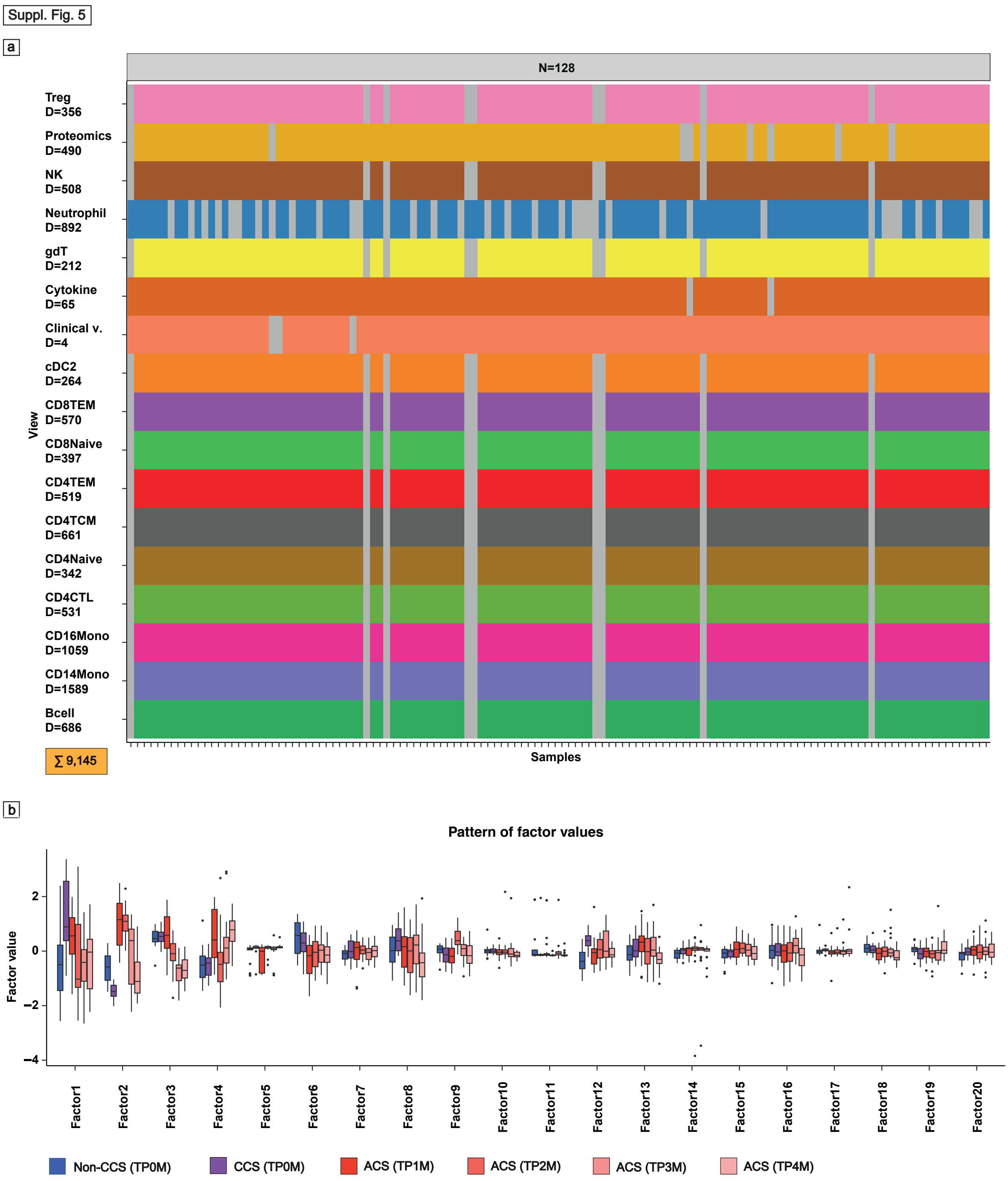

Suppl. Fig. 5

(a) Overview data input MOFA with automated azimuth annotations (exploratory Munich cohort): amount of features for each input data dimension (D), amount of samples (N, defined as combinations of subjects and timepoints TP1M/TP2M/TP3M/TP4M) and missing data highlighted by grey colors (specifying missing data for a specific dimension and sample). **(b)** Factor values of all samples (n=128) in longitudinal comparison for all 20 MOFA factors

estimated with azimuth annotations and input views as visualized in (a). Data are shown as a Box-Whiskers plot (box: median, 25th to 75th percentile; whiskers: hinge to largest/ smallest value, no further than 1.5*IQR from hinge).

Suppl. Fig. 6

a

Sample factor values

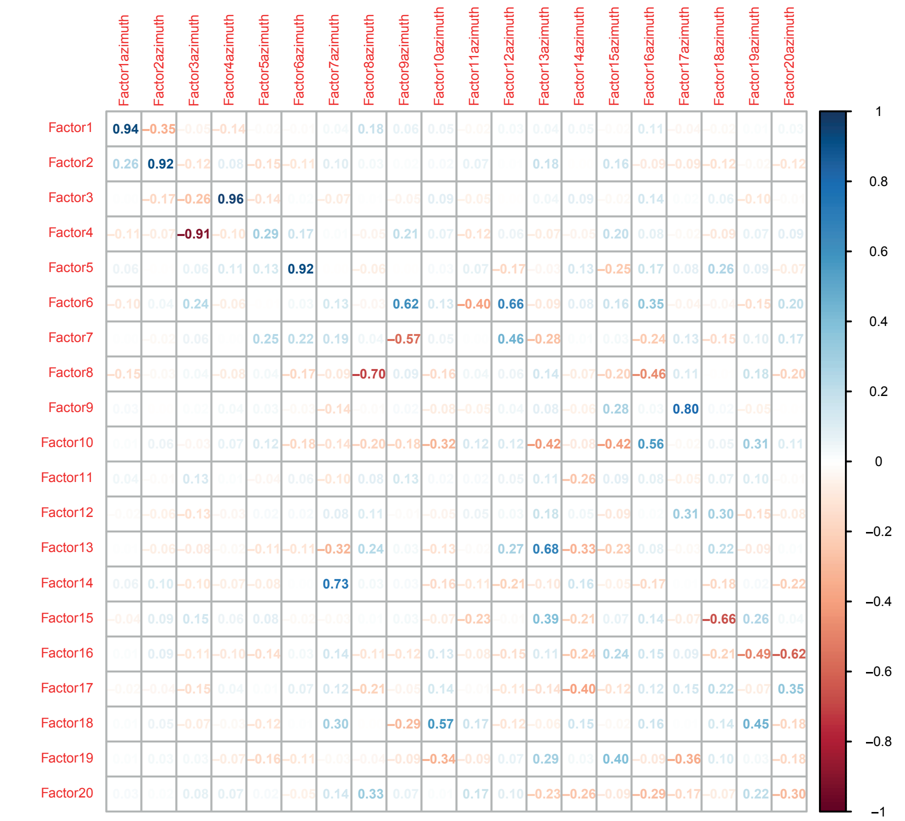

b

Feature factor weights

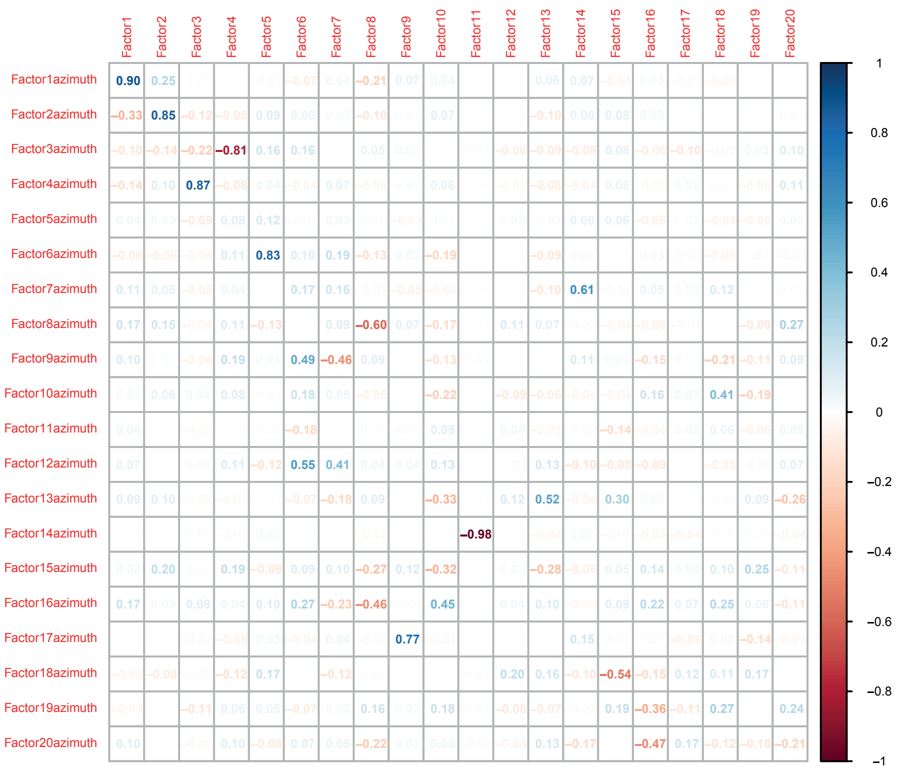

Suppl. Fig. 6

(a) Pearson correlation between **sample factor values** inferred by two different MOFA models: (1) model with manual cell-type cluster annotation (Suppl. Fig. 3) (y-axis) and (2) model with automated azimuth annotations (Suppl. Fig. 5) (x-axis). **(b)** Pearson correlation between **feature factor weights** inferred by two different MOFA models; (1) model with automated azimuth annotations (Suppl. Fig. 5) (y-axis) and (2) model with manual cell-type cluster annotation (Suppl. Fig. 3) (x-axis).

Note: in order to correlate features for Suppl. Fig 6b based on the different cell-type annotation levels given by model (1) and model (2) we mapped features as given below:

**Munich approach:**<br>**clusters and annotations**	**Groningen approach:**<br>**automated azimuth**
CD14^{high} monocytes clusters (cluster 4, 6, 7)	CD14 Mono
B cells (cluster 8, 10)	B cell (aggregating ‘B naive', ‘B memory’, ‘B intermediate’)
CD16^{high} monocytes (cluster 9, 12)	CD16 Mono
CD4^{+} T cells (cluster 0, 2)	CD4TCM
Dendritic cells (cluster 13)	cDdC2
NK cells (cluster 3)	NK
CD4^{+} T cells (cluster 5, 11)	No mapping/
CD8^{+} T cells (cluster 1)	No Mapping /

The left column of the table gives the cluster number and annotation of the cluster of the initial manual marker gene annotation strategy. The right column shows the most similar azimuth-based annotation that could be mapped to this cluster based on a high overlap of cells between the corresponding azimuth cell-type and marker gene-based cluster annotation (Suppl. Fig. 4a).

As an example: a specific gene from cluster 4 $CD14^{high}$ monocytes (e.g. *HMGB1*) has been mapped to the same gene (e.g. *HMGB1*) of the aggregated CD14 Mono dimension given by the automated azimuth annotation, then the weights across all features have been correlated. Features from cluster 1, 5 and cluster 11 were not mapped due to the low overlap of cells between the different annotation strategies and therefore excluded from the correlation evaluation presented in Suppl. Fig. 6b.

Suppl. Fig. 7

a

Sample factor values

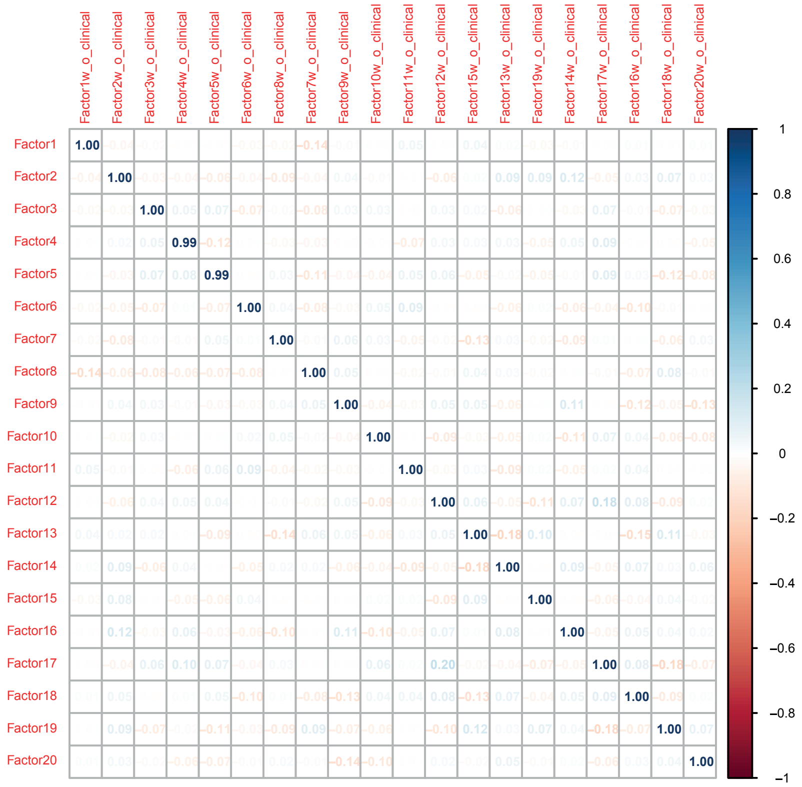

b

Feature factor weights

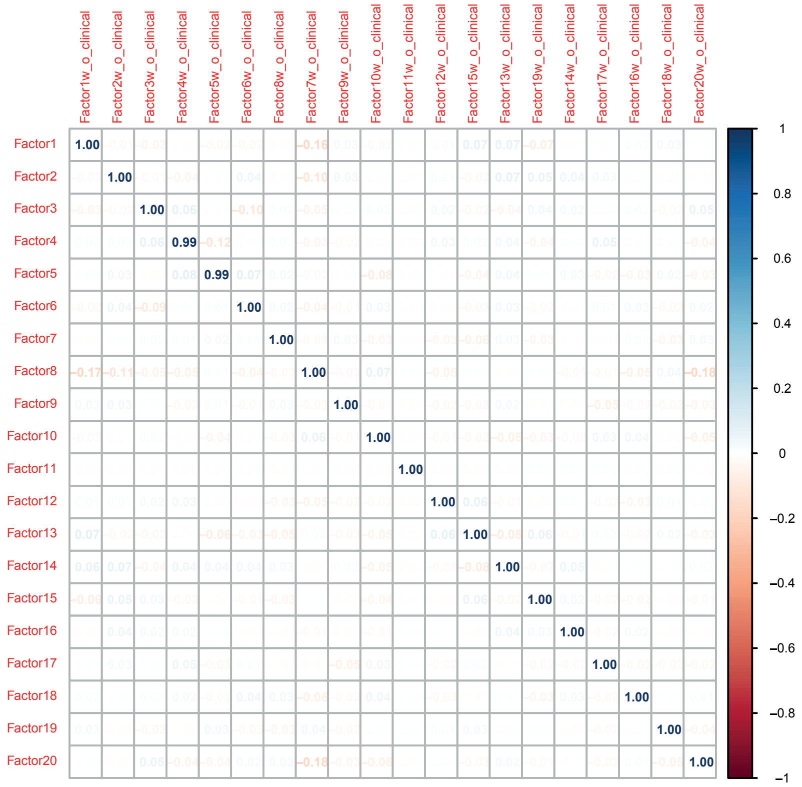

Suppl. Fig. 7

(a) Pearson correlation between **sample factor values** inferred by two different MOFA models: (1) model including clinical features (y-axis) as visualized in Suppl. Fig. 3 and (2) model without clinical features (x-axis). **(b)** Pearson correlation between **feature factor weights** inferred by two different MOFA models; (1) model including clinical features (y-axis) as visualized in Suppl. Fig. 3 and (2) model without clinical features (x-axis) per factor.

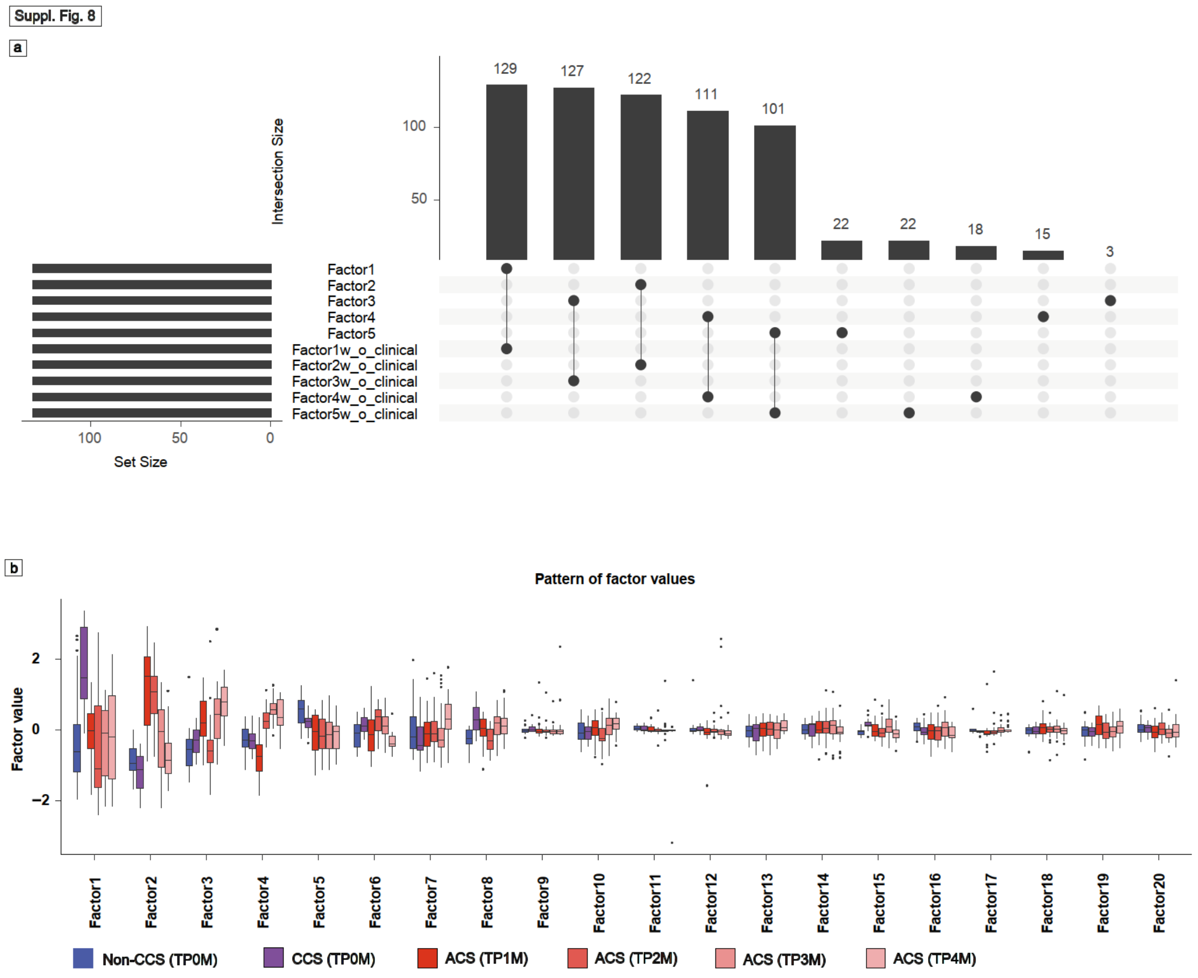

Suppl. Fig. 8

(a) Comparison of the overlap of top 1% of features (D=132) between MOFA model inferred with clinical variables as features and without clinical variables (w_o_clinical). **(b)** Factor values of all samples (n=128) in longitudinal comparison for all 20 MOFA factors inferred by the MOFA model trained without clinical features. Data are shown as a Box-Whiskers plot (box: median, 25th to 75th percentile; whiskers: hinge to largest/ smallest value, no further than 1.5*IQR from hinge).

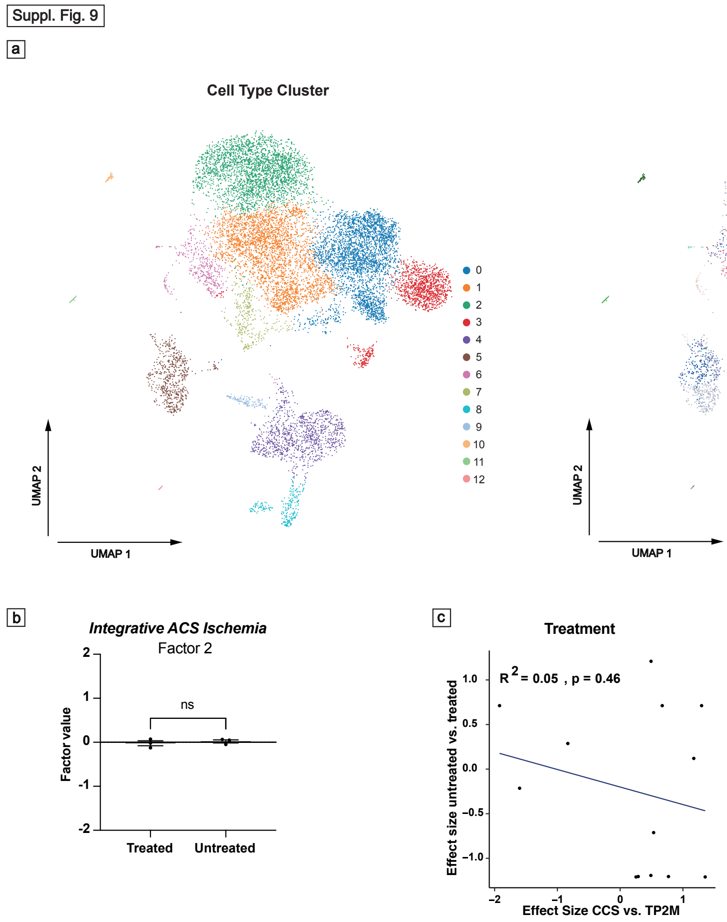

Suppl. Fig. 9

(a) UMAPs of scRNA-seq data from IV PBMCs of samples treated with medication (n=3) and without medication (n=3), showing cells colored by different identified cell clusters (left) and colored by automated cell-type annotation resulting from the Azimuth annotation pipeline (right) (n=12,994). **(b)** *Integrative ACS Ischemia* (Factor 2) computed on the IV PBMC data. Comparison of the factor values of treated (n=3) and untreated (n=3) samples. The parametric dataset was analyzed using the paired t-test (two-sided, p-value: 0.3395). ns p>0.05. The y-axis was adjusted to the size of the IAI scale. **(c)** Scatterplot visualizing relationship (R^2 = squared pearson correlation coefficient; two-sided t-test) of effect sizes of high-ranking *Integrative ACS Ischemia* genes visualized in main figures by violin plots (CD14.Mono: *VCAN, SOCS3, PSME2, ODC1, JAK1, IL6ST, CD74; CD4.TCM: UBC, STAT3, JAK1, IL6ST, HMGB1, HINT1, EIF3E*) based on the corresponding azimuth annotation. Effect sizes (β) were calculated using a linear model for the comparison of patients with CCS (TP0M n=16) vs patients with sterile ACS at TP2M (n=19) in the pseudobulk aggregated CS scRNA-seq data

and untreated (U n=3) vs. treated (T n=3) patients in the pseudobulk aggregated IV scRNA-seq data (see methods).

Suppl. Fig. 10

a

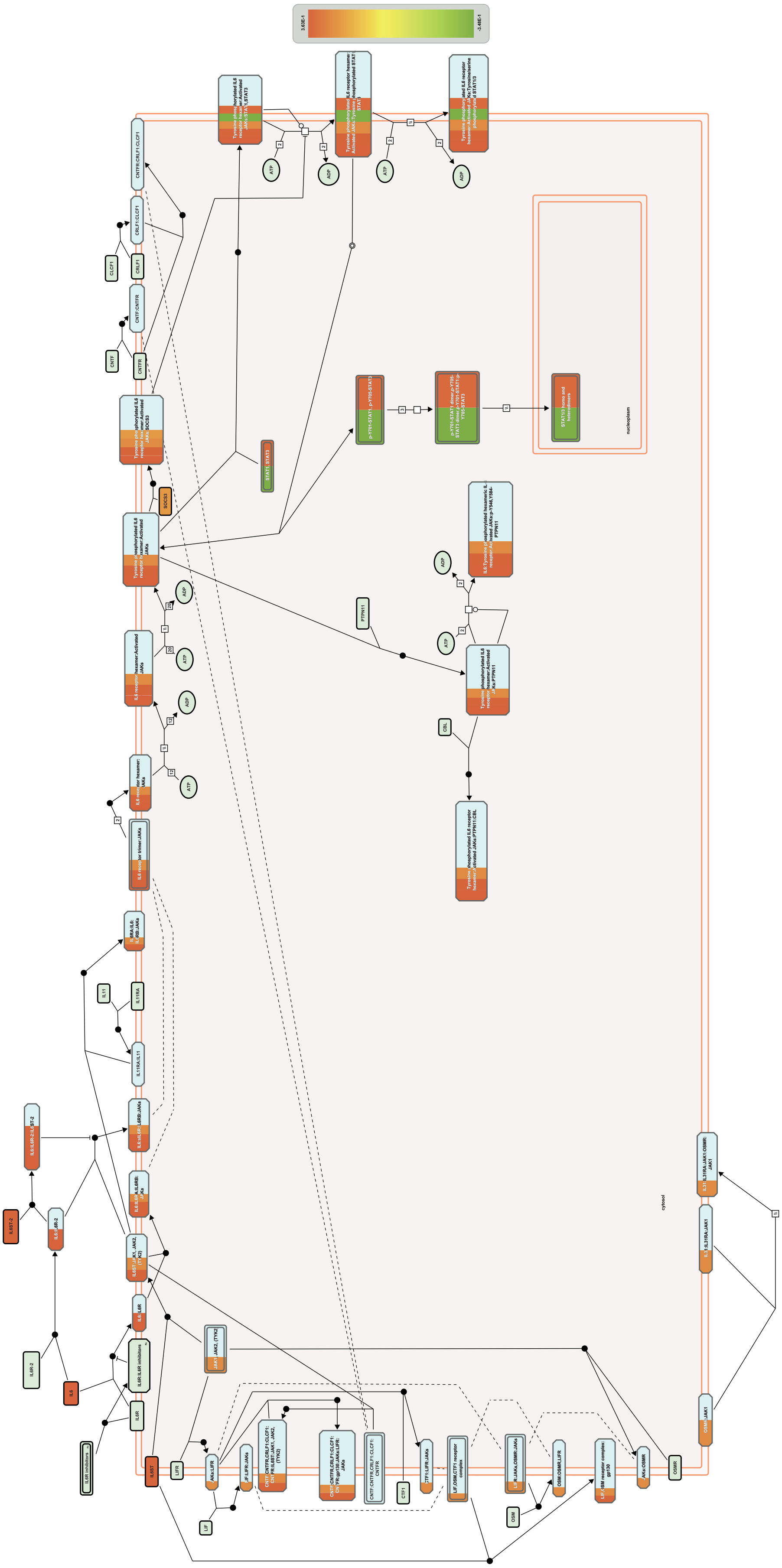

Suppl. Fig. 10

(a) The components of the cellular interleukin-6 cascade illustrated by REACTOME. The coloring of the components is based on the average factor values of the genes belonging to the pathway and the top 25% of genes on *Integrative ACS*. Adapted from URL: https://reactome.org/content/detail/R-HSA-1059683

Suppl. Fig. 11

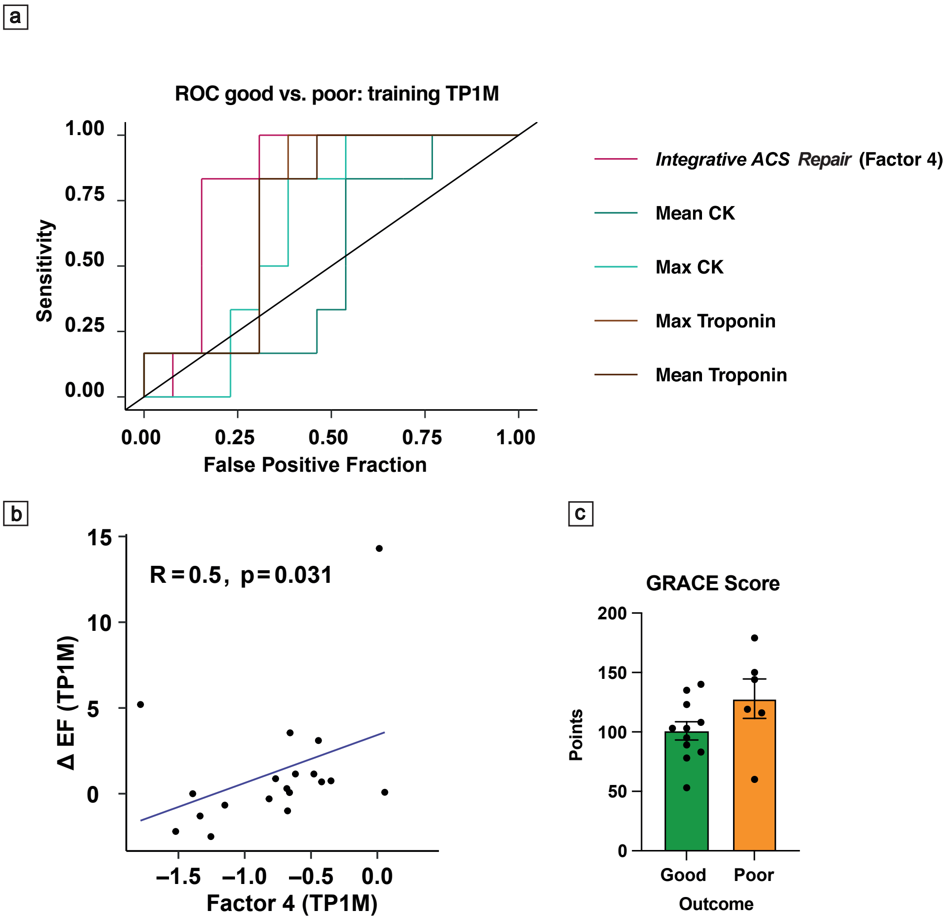

Suppl. Fig. 11

(a) ROC AUC. Comparison of the predictive power of *Integrative ACS Repair* (Factor 4) (n=19) and mean and max CK levels (n=19) and mean and max CRP troponin levels (n=19) based on the complete longitudinal values. **(b)** Spearman correlation of *Integrative ACS Repair* (Factor 4) values for samples at TP1M and measured delta ejection fraction (ΔEF) values (n =19) (two-sided t-test). **(c)** Risk stratification using the Grace Score. Comparison of the good (M, n=11) and poor outcome (M, n=6) group based on the assessment of the Grace Score. The parametric dataset was analyzed using an unpaired t-test (two-sided). Exact p-values were summarized in Suppl. Table 13. *p≤0.05. Mean values with +/- SEM are shown.

Suppl. Fig. 12

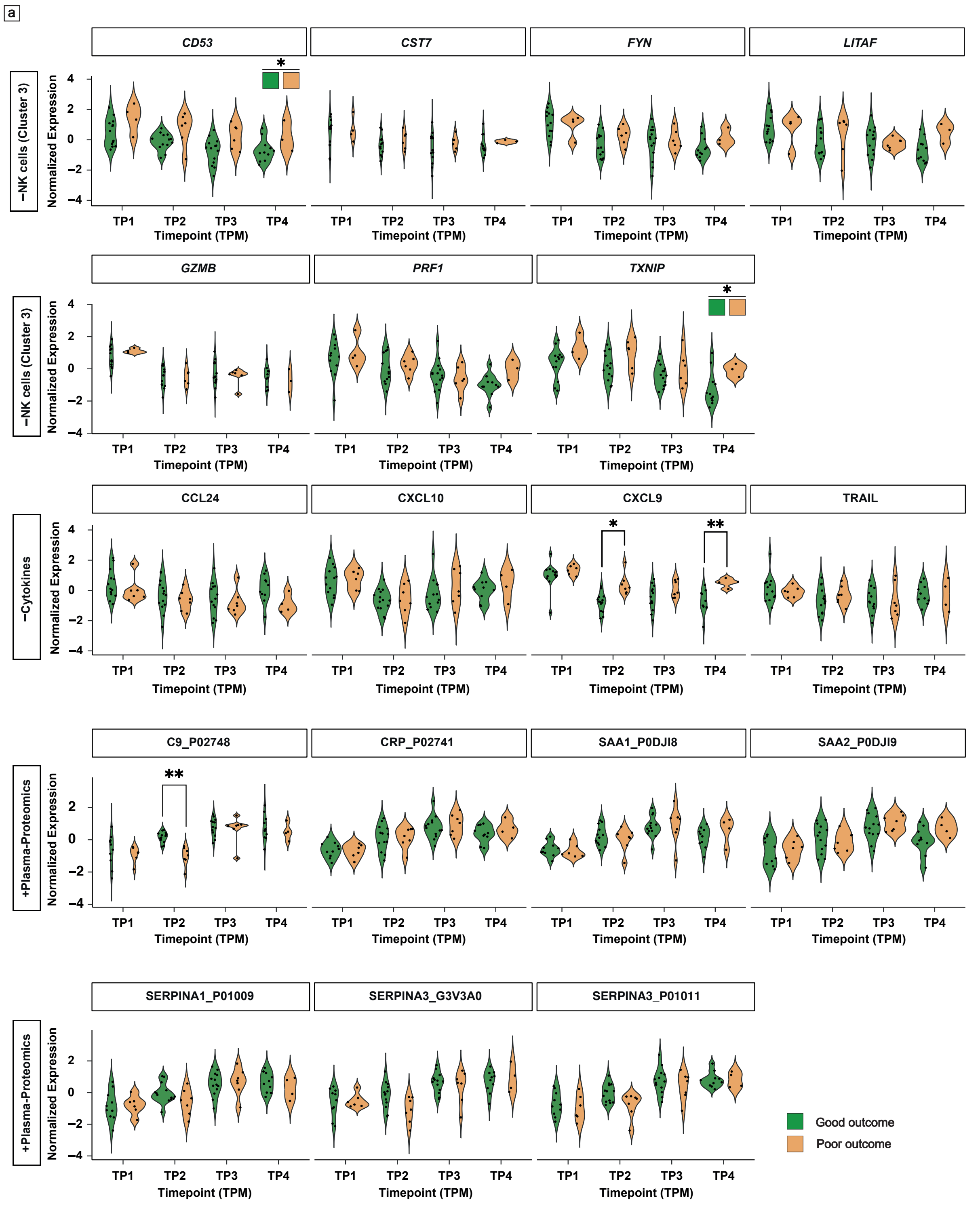

Suppl. Fig. 12

(a) Normalized expression values of selected top features of *Integrative ACS Repair* (Factor 4) in longitudinal comparison for samples classified with good or poor outcome ((NK cells (cluster 3): good outcome: TP1M n=13, TP2M n=13, TP3M n=14, TP4M=11; poor outcome: TP1M n=4, TP2M n=6, TP3M n=6, TP4M n=3) (Cytokines: good outcome: TP1M n=13, TP2M

n=14, TP3M n=13, TP4M=10; poor outcome: TP1M n=6, TP2M n=7, TP3M n=7, TP4M n=4) (Plasma-Proteomics: good outcome: TP1M n=10, TP2M n=13, TP3M n=13, TP4M=10; poor outcome: TP1M n=6, TP2M n=7, TP3M n=7, TP4M n=4)). The dataset was analyzed using the Mixed-effects analysis with correction for multiple comparisons by Šidák test. In case only the column factor was significant, graphs are marked with a vertical bar on top. *$p \leq 0.05$, **$p \leq 0.05$. (+/- indicates the direction of the feature factor weight). Exact p-values were summarized in Suppl. Table 13.

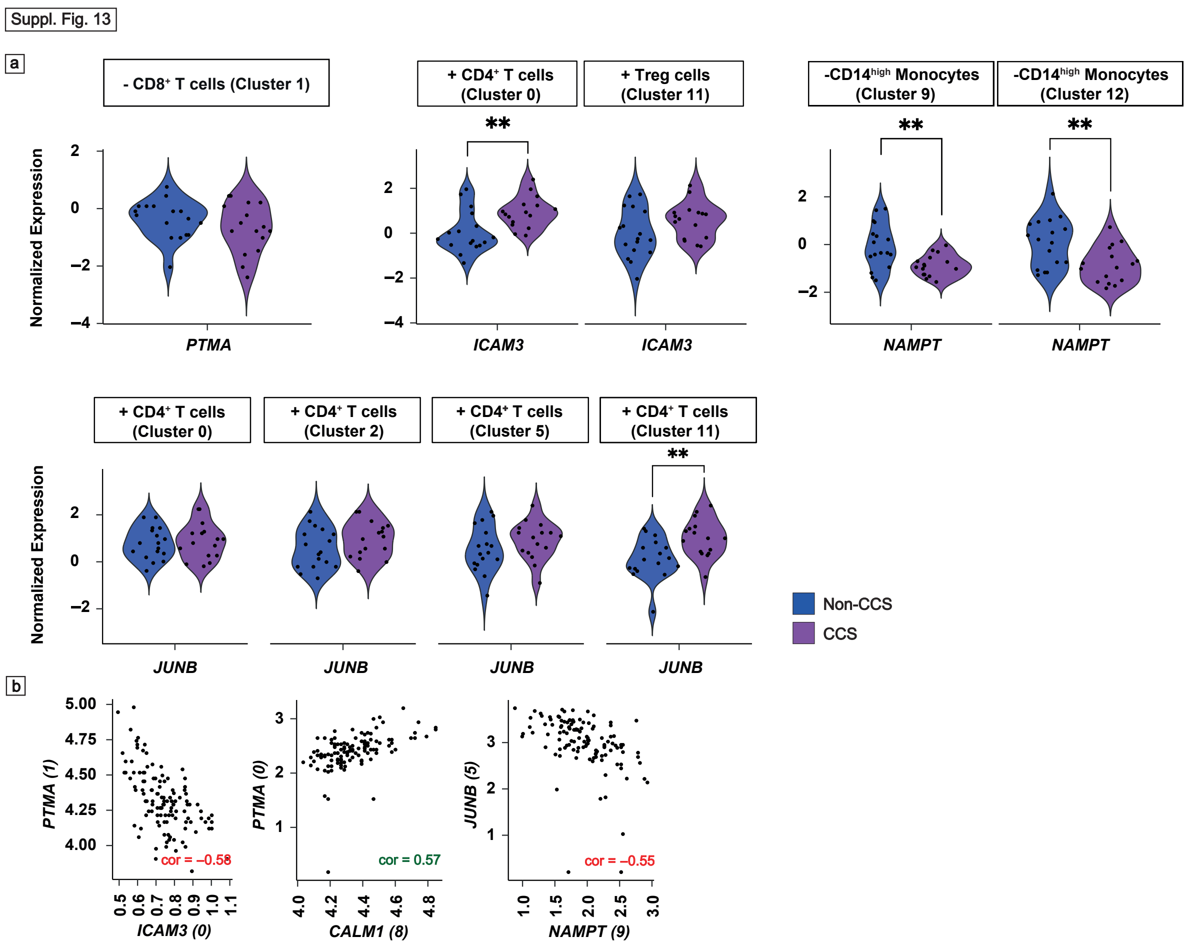

Suppl. Fig. 13

(a) Normalized expression values of selected top features of *Integrative CCS* (Factor 1) in comparison for samples classified CCS (TP0M n=16) and non-CCS (TP0M n=17)**.** Parametric data were analyzed using an unpaired t-test (two-sided). *p≤0.05, **p≤0.01. (+/- indicates the direction of the feature factor weight). Exact p-values were summarized in Suppl. Table 13. **(b)** Correlation score of selected examples of circoplots (a,b).

Suppl. Fig. 14

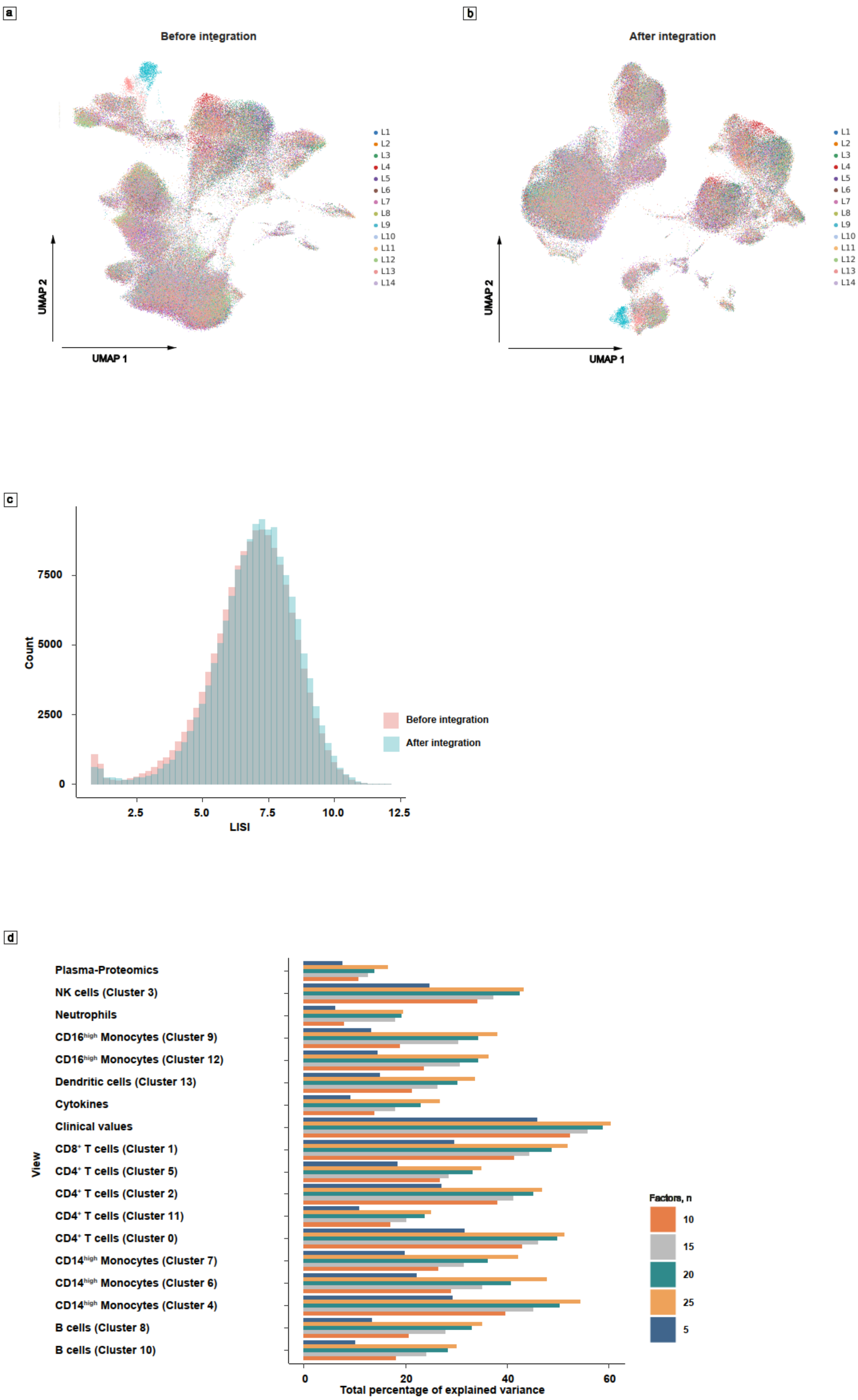
a
Before integration
UMAP 2
UMAP 1
L1
L2
L3
L4
L5
L6
L7
L8
L9
L10
L11
L12
L13
L14
b
After integration
UMAP 2
UMAP 1
L1
L2
L3
L4
L5
L6
L7
L8
L9
L10
L11
L12
L13
L14
c
Count
7500
5000
2500
0
2.5
5.0
7.5
10.0
12.5
LISI
Before integration
After integration
d
View
Plasma-Proteomics
NK cells (Cluster 3)
Neutrophils
CD16high Monocytes (Cluster 9)
CD16high Monocytes (Cluster 12)
Dendritic cells (Cluster 13)
Cytokines
Clinical values
CD8+ T cells (Cluster 1)
CD4+ T cells (Cluster 5)
CD4+ T cells (Cluster 2)
CD4+ T cells (Cluster 11)
CD4+ T cells (Cluster 0)
CD14high Monocytes (Cluster 7)
CD14high Monocytes (Cluster 6)
CD14high Monocytes (Cluster 4)
B cells (Cluster 8)
B cells (Cluster 10)
0
20
40
60
Total percentage of explained variance
Factors, n
10
15
20
25
5

Suppl. Fig. 14

(a) UMAP of scRNA-seq data from PBMCs showing cells before integration with Scanorama colored by the library of origin (L1-L14). **(b)** UMAP of scRNA-seq data from PBMCs showing cells after integration with Scanorama colored by the library of origin (L1-L14). **(c)** Comparison of LISI score distribution before integration and after integration with Scanorama. **(d)** Total percentage of explained variance (x-axis) for each input view (y-axis) of MOFA models with different number of estimated factors (5,10,15,20,25).
