# Supplementary material for: Multiomic analyses uncover immunological signatures in acute and chronic coronary syndromes
Source: Nat Med. 2024 May 21;30(6):1696–710. doi: 10.1038/s41591-024-02953-4 (PMC11186793; doi:10.1038/s41591-024-02953-4)
Supplement: Supplementary file 2 — Reporting Summary [file 41591_2024_2953_MOESM2_ESM.pdf]

nature portfolio

Corresponding author(s): Dr. Kami Pekayvaz, Dr. Matthias Heinig, Dr. Leo Nicolai, Prof. Dr. Konstantin Stark

Last updated by author(s): 10/03/2024

Reporting Summary

Nature Portfolio wishes to improve the reproducibility of the work that we publish. This form provides structure for consistency and transparency in reporting. For further information on Nature Portfolio policies, see our Editorial Policies and the Editorial Policy Checklist.

Statistics

For all statistical analyses, confirm that the following items are present in the figure legend, table legend, main text, or Methods section.

n/a	Confirmed	
☐	☒	The exact sample size (*n*) for each experimental group/condition, given as a discrete number and unit of measurement
☐	☒	A statement on whether measurements were taken from distinct samples or whether the same sample was measured repeatedly
☐	☒	The statistical test(s) used AND whether they are one- or two-sided<br>*Only common tests should be described solely by name; describe more complex techniques in the Methods section.*
☐	☒	A description of all covariates tested
☐	☒	A description of any assumptions or corrections, such as tests of normality and adjustment for multiple comparisons
☐	☒	A full description of the statistical parameters including central tendency (e.g. means) or other basic estimates (e.g. regression coefficient) AND variation (e.g. standard deviation) or associated estimates of uncertainty (e.g. confidence intervals)
☐	☒	For null hypothesis testing, the test statistic (e.g. *F*, *t*, *r*) with confidence intervals, effect sizes, degrees of freedom and *P* value noted<br>*Give P values as exact values whenever suitable.*
☒	☐	For Bayesian analysis, information on the choice of priors and Markov chain Monte Carlo settings
☒	☐	For hierarchical and complex designs, identification of the appropriate level for tests and full reporting of outcomes
☐	☒	Estimates of effect sizes (e.g. Cohen's *d*, Pearson's *r*), indicating how they were calculated

Our web collection on statistics for biologists contains articles on many of the points above.

Software and code

Policy information about availability of computer code

Data collection	Data collection and analysis were performed alongside, summarized below.
Data analysis	The statistical analysis and the graphical illustration of clinical values, cytokine measurements and some graphical illustrations of the Multi-Omic Factors were performed with GraphPad Prism (version: 9.2.0 or 10.0.3).<br>For proteomics data, raw files were analysed in Spectronaut 14 (Biognosys) against a spectral library that was generated from 52 fractions measured in the same manner as described in the linked literature. An FDR cutoff of 0.01 was applied and spectra were searched against a human Uniprot database from 2018 including isoforms. For data filtering, the option Qvalue percentile with a fraction of 0.2 was used and global normalisation by median was applied. Further downstream analysis was performed in R. Normalised intensities were filtered for at least 80% valid values per row and column, remaining missing values were median-centred and imputed using a randomised Gaussian distribution with a downshift of 1.8. For significance calling, the limma package was consulted to calculate moderated t statistics, described in the cited literature. Nominal p-values were corrected using the Benjamini-Hochberg method.<br>For bulk RNA-seq analysis, the following analysis was performed: The sequencing reads were processed using zUMIs pipeline using the Gencode human release version (https://www.gencodegenes.org/human/release_35.html). Only barcodes matching the expected samples were considered and exported as count matrices, both raw counts and library-size normalized ones. First, the data was checked using fastqc (version 0.11.8). Regions on the 3' end of the fragment reading into the poly-A tail were removed by Cutadapt (version 1.12). The zUMIs pipeline (version 2.9.4d) was applied, filtering the data, with a phred threshold of 20 for 4 bases the UMI and BC, mapping the reads to the human genome with the Gencode annotation (v35) using STAR (version 2.7.3a), reads were counted using RSubread (version 1.32.4).<br>Flow cytometry data was analysed with the following software: The flow cytometry data were analysed with FlowJo (BD, version: 10.8.1). The statistical analysis and the graphical illustration were performed with GraphPad Prism (version: 9.2.0).<br>Figure alignement was performed by Adobe Illustrator (version: 25.4.1).<br>QC and processing of the scRNA-seq data as well as the MOFA analysis were performed in R (version: 4.1.1) and Python (version: 3.9.6) using

the following packages:
Python: scanpy (version: 1.8.1) ; scanorama (version: 1.7.1)
R: Seurat (version: 4.1.1); MOFA2 (version: 1.2.2); clusterProfiler (version 4.0.5); nichenetr (version: 1.1.0) ; Azimuth (version: 0.4.6); reactome.db (version: 1.76.0); ReactomePA (version: 1.36.0);
glmnet (version: 4.1.6). LEGENDplex™ data were analysed using BioLegend's LEGENDplex™ data analysis software (Analysed with newest version in September 2023).
All code will is available on github at https://github.com/heiniglab/stemi_mofa

For manuscripts utilizing custom algorithms or software that are central to the research but not yet described in published literature, software must be made available to editors and reviewers. We strongly encourage code deposition in a community repository (e.g. GitHub). See the Nature Portfolio guidelines for submitting code & software for further information.

Data

Policy information about availability of data

All manuscripts must include a data availability statement. This statement should provide the following information, where applicable:

- Accession codes, unique identifiers, or web links for publicly available datasets
- A description of any restrictions on data availability
- For clinical datasets or third party data, please ensure that the statement adheres to our policy

All mapped data (bulk proteomics, cytokine measurements, biomarkers of inflammation and myocardial damage, ejection fraction, bulk and single-cell RNA-seq count matrices), from the Munich cohort is available from Zenodo upon approval for immunological research purposes (DOI: 10.5281/zenodo.10815146). Processed pathway annotations and auxiliary data is also provided on Zenodo. Raw sequencing data of the Groningen cohort is available from the European Genome-Phenome Archive https://ega-archive.org/datasets/EGAD00001010064 and processed data is available from https://eqtlgen.org/sc/datasets/blokland2024-dataset.html . Ligand receptor data and regulatory potential scores from the NicheNet model21 were downloaded from https://zenodo.org/record/3260758/files/lr_network.rds (ligand receptor network) and https://zenodo.org/record/3260758/files/ligand_target_matrix.rds (regulatory potential scores).
Reference data for the azimuth mapping was a previously annotated and published multimodal CITE-seq (combined scRNA-seq and protein expression) reference dataset of 162,000 PBMCs67.

Research involving human participants, their data, or biological material

Policy information about studies with human participants or human data. See also policy information about sex, gender (identity/presentation), and sexual orientation and race, ethnicity and racism.

Reporting on sex and gender	Gender and sex was self reported, no gender or sex-matched sub-analyses were performed. Samples were not included based on gender or sex, the distribution was random based on patient presentation to the hospital.
Reporting on race, ethnicity, or other socially relevant groupings	Race, ethnicity or other socially relevant grouping was not included into the manuscript.
Population characteristics	In the Munich cohort n=11 healthy controls (non-CCS, median age 55y), n=7 patients with coronary sclerosis (non-CCS, median age 66y), n=16 patients with CCS (median age 64y) or with n=28 ACS (median age 61y) were included. In the Groningen cohort (external dataset, reanalyzed) n=31 control subjects (median age 58y) and n=24 patients (median age 64y) were included.<br>No further age, sex, or gender-based subanalyses were planned since the study-design and group size was anticipated to not allow for these sub-group analyses.
Recruitment	By self-referal or physician referal. Patients presenting to the LMU Klinikum with STEMI were included into the study when meeting the study criteria and consenting. There was no specific bias in patient selection.
Ethics oversight	For the Munich cohort: Ethics Committee of the Ludwig Maximilian University of Munich (No.: 19-274)<br>For the Groningen cohort, details are provided in the original manuscript: https://doi.org/10.1101/2023.05.02.23289370

Note that full information on the approval of the study protocol must also be provided in the manuscript.

Field-specific reporting

Please select the one below that is the best fit for your research. If you are not sure, read the appropriate sections before making your selection.

☒ Life sciences ☐ Behavioural & social sciences ☐ Ecological, evolutionary & environmental sciences

For a reference copy of the document with all sections, see nature.com/documents/nr-reporting-summary-flat.pdf

Life sciences study design

All studies must disclose on these points even when the disclosure is negative.

Sample size	The sample sizes used in this exploratory study were aligned to previous single-cell based profiling studies or functional in vitro assays, the total sample size exceeds most available single-cell based immune-cell profiling studies to date. Example studies on which sample size

	estimations were based on were the following: PMID (32669297, 32747814, 34174187, 34352228, 32807934, 32991843, 32783921, 33398161, 35803260, 32103181, 36624340)
Data exclusions	Sample exclusion from multi-omics or experimental data was performed prior to further integration and analysis, if the respective samples failed standardized quality control. In the Groningen cohort only V2 chemistry samples were included. Since v3 10x chemistry showed technical differences in gene expression profiles between the samples that were prepared with different chemistries a separate processing of both datasets was necessary. In our replication we focused on samples measured with the v2 10X chemistry as this cohort included a higher number of samples (v2: n=55; v3: n=21). Additionally, the V3 10X chemistry cohort did not include a sufficient number of samples which could be divided into poor or good outcome groups and would have therefore been underpowered.
Replication	An independent study cohort was employed as included in the manuscript, which reproduced key findings of the manuscript.
Randomization	Randomization was not performed because this would be unethical. All patient received optimal, guideline-guided treatment.
Blinding	Most analyses were performed in an unsupervised manner. During flow-cytometry analyses or functional assays investigators were not blinded concerning sample cohorts (to allow sample identification) but were blinded concerning exact hypotheses. Quantification was based on unbiased analysis strategies (such as gating).

Reporting for specific materials, systems and methods

We require information from authors about some types of materials, experimental systems and methods used in many studies. Here, indicate whether each material, system or method listed is relevant to your study. If you are not sure if a list item applies to your research, read the appropriate section before selecting a response.

Materials & experimental systems

n/a	Involved in the study
☐	☒ Antibodies
☒	☐ Eukaryotic cell lines
☒	☐ Palaeontology and archaeology
☒	☐ Animals and other organisms
☐	☒ Clinical data
☒	☐ Dual use research of concern
☒	☐ Plants

Methods

n/a	Involved in the study
☒	☐ ChIP-seq
☐	☒ Flow cytometry
☒	☐ MRI-based neuroimaging

Antibodies

Antibodies used	Antibodies for flow cytometry:<br>BV650 anti-human CD45 Antibody (BioLegend, cat#304044, clone: HI30);<br>FITC anti-human CD14 Antibody (BD Pharmingen™, cat#557153, clone: M5E2);<br>PE anti-human CD16 Antibody (BioLegend, cat#302008, clone: 3G8);<br>PerCP-Cyanine5.5 anti-human CD8 Antibody (BioLegend, cat#344710, clone: SK1);<br>APC-Cy7 anti-human CD19 Antibody (BioLegend, cat#363010, clone: SJ25C1);<br>BV605 anti-human HLA-DR Antibody (BioLegend, ca#307640, clone: L243);<br>BV785 anti-human CD123 Antibody (BioLegend, cat#306032, clone: 6H6);<br>BV510 anti-human CD3 Antibody (BioLegend, cat#317332, clone: OKT3);<br>BV711 anti-human CD56 Antibody (BioLegend, cat#362542, clone: 5.1H11);<br>AF700 anti-human CD34 Antibody (BioLegend, cat#343622, clone: 561);<br>BV421 anti-human CD4 Antibody (BioLegend, cat#357424, clone: A161A1);<br>SytoxRed Dead cell stain (Invitrogen, cat#S34859);<br>V500 anti-human CD14 M5E2 (RUO) BD Biosciences cat#561391;<br>PE anti-human CD93 VIMD2 BioLegend cat#336108;<br>PerCP-Cy5.5 anti-human CD36 5-271 BioLegend cat#336224;<br>APC anti-human SLAN REA1050 Miltenyi Biotec cat#130-117-919;<br>APC/Fire™ 750 anti-human CD88 S5/1 BioLegend cat#344316;<br>BV605 anti-human HLA-DR L243 BioLegend cat#307640;<br>BV785 anti-human CD192 K036C2 BioLegend cat#357234;<br>BV510 anti-human CD16 3G8 BioLegend cat#302048;<br>BV711 anti-human CD86 IT2.2 BioLegend cat#305440;<br>AF700 anti-human CD89 A59 BioLegend cat#354118;<br>PE/Dazzle anti-human CD11a HI111 BioLegend cat#301232;<br>PE-Cy7 anti-human CD9 HI9a BioLegend cat#312116;<br>SytoxBlue Dead cell stain Invitrogen cat#S34857;<br>AF700 anti-human CD4 A161A1 Biolegend cat#357418;<br>BV605 anti-human CD8 SK1 Biolegend cat#344742;<br>BV785 anti-human CD45RO UCHL1 Biolegend cat#304234;<br>BV421 anti-human CCR7 G043H7 Biolegend cat#353208;<br>APC anti-human PD1 EH12.2H7 Biolegend cat#329908;<br>PE-Cy7 anti-human CD69 FN50 Biolegend cat#310912;

PE-Dazzle594 Canti-human D366 (Tim3) F38-2E2 Biolegend cat#345034;

Hashtag for scRNA-seq:
TotalSeq™-B0251 anti-human Hashtag 1 (BioLegend, cat#394631, clone: LNH-94; 2M2);
TotalSeq™-B0252 anti-human Hashtag 2 (BioLegend, cat#394633, clone: LNH-94; 2M2);
TotalSeq™-B0253 anti-human Hashtag 3 (BioLegend, cat#394635, clone: LNH-94; 2M2);
TotalSeq™-B0254 anti-human Hashtag 4 (BioLegend, cat#394637, clone: LNH-94; 2M2);
TotalSeq™-B0255 anti-human Hashtag 5 (BioLegend, cat#394639, clone: LNH-94; 2M2);
TotalSeq™-B0256 anti-human Hashtag 6 (BioLegend, cat#394641, clone: LNH-94; 2M2);
TotalSeq™-B0257 anti-human Hashtag 7 (BioLegend, cat#394643, clone: LNH-94; 2M2);
TotalSeq™-B0258 anti-human Hashtag 8 (BioLegend, cat#394645, clone: LNH-94; 2M2);
TotalSeq™-B0259 anti-human Hashtag 9 (BioLegend, cat#394647, clone: LNH-94; 2M2);
TotalSeq™-B0260 anti-human Hashtag 10 (BioLegend, cat#394649, clone: LNH-94; 2M2)

Validation

Reported on the respective manufacturer`s websites, i.e. Biolegend states on their website (https://www.biolegend.com/nl-nl/bio-bits/highly-specific-validated-antibodies) that antibodies are tested on different cells/tissues, expression levels and applications for ensuring sensitivity and specificity. BD Biosciences states (https://www.bdbiosciences.com/en-us/products/reagents/flow-cytometry-reagents/research-reagents/quality-and-reproducibility) similarly that antibodies are tested on a combination of cells/cell lines or transfectant models by different methods. Miltenyi Biotec includes a similar statement on their website (https://www.miltenyibiotec.com/DE-en/products/macs-antibodies/antibody-validation.html).

Clinical data

Policy information about clinical studies
All manuscripts should comply with the ICMJE guidelines for publication of clinical research and a completed CONSORT checklist must be included with all submissions.

Clinical trial registration

N/A

Study protocol

Observational study

Data collection

Informed consent was obtained from the patients in accordance with the Declaration of Helsinki and with the approval of the Ethics Committee of the Ludwig Maximilian University of Munich (No.: 19-274). We collected blood from n=62 patients employing repetitive serial sampling and separately analysed the different immune cell constituents. For blood collection we used heparin-anticoagulated blood (i.e. Sarstedt AG & Co. KG, cat#02.1065.001).
In the acute coronary syndrome (ACS) group, patients with ST segment elevation myocardial infarction (STEMI) were included and blood was analysed longitudinally. Blood sampling was done periinterventionally (TP1M) – during catheterization to avoid time loss, 14 (± 8) h after intervention (TP2M), 60 (± 12) h after acute event (TP3M) and before discharge, about 5-8 d after acute event (TP4M). A further subdivision was made into patients without direct reperfusion within 24h after symptom onset (delayed myocardial reperfusion, n=4) and patients with direct reperfusion within 24h due to coronary intervention (acute myocardial infarction, n=24). A subgroup of patients with evidence of infection in laboratory testing who were treated with antibiotics in the clinical setting defined a subgroup with hospital acquired infection (n=5) which was differentiated from the sterile group with STEMI without hospital acquired infections (n=19). The latter was used for comparison with the chronic coronary syndrome group (CCS).

Outcomes

For this purpose, the ejection fraction (EF) measurement was determined according to Simpson's method in echocardiography. A comparison was made between the findings on admission and during the hospital stay or before discharge (ΔEF). Based on these, a classification was made according to positive (good outcome) and negative (poor outcome) ΔEF in acute setting. The stratification of the Groningen cohort was performed in a similar manner, however with a longer time-period in-between initial and secondary measurement.
The CCS group included patients with an initial diagnosis of chronic coronary syndrome based on a cardiac catheterisation (lumen reduction of >50%) or coronary CT scan (>75 percentile) (CCS, TP0M n=16). Coronary healthy patients, with a catheter or CT based rule out of CAD, were included as a comparison group for the chronic coronary syndrome group (non-CCS, TP0M n=18). Coronary sclerosis was defined as coronary irregularities without significant lumen obstruction (<50%). For ACS outcome prediction the GRACE Score was used (PMID: 24561498 and 17032691).

Methodology

Sample preparation

Peripheral blood mononuclear cells (PBMCs) were isolated from whole blood by using a BD Vacutainer® CPTTM (Becton, Dickinson and Company, cat#362780). After centrifugation (1650 x g for 20 min at room temperature (RT) (Centrifuge 5810 R, Eppendorf AG)), the supernatant was transferred into a new tube and was followed by a further centrifugation step (350 x g for 7 min at 4°C). The resulting cell pellet was resuspended in 4 ml freezing medium (45% RPMI (VLE-RPMI 1640, Bio&SELL GmbH, cat#BS.52551528.5) with 1% glutamine (GibcoTM L-Glutamine (200 mM), Thermo Fisher Scientific, cat# BS.K0283), 45% FBS (FBS SUPERIOR stabil®, Bio&SELL GmbH, cat#FBS.S0615) and 10% DMSO (Dimethyl sulphoxide, Sigma-Aldrich Chemie GmbH, cat#D2438)). For cryoasservation, samples were slowly frozen in a Mr. Frosty freezing container (Thermo Fisher Scientific, cat#5100-0036) at -80°C for 24h and then transferred to -80°C freezers.
For scRNA-seq analysis and flow cytometry of the frozen PBMCs, an adapted thawing protocol of 10X was used (Fresh Frozen Human Peripheral Blood Mononuclear Cells for Single Cell RNA Sequencing, Document Number CG00039 Rev E, 10x Genomics, (2023, May 2nd): https://assets.ctfassets.net/an68im79xiti/71r5PbRPB1LeqRkuPltBzr/64cfaa099d0a7fd41f79a4aecd643926/CG00039_Demonstrated_Protocol_FreshFrozenHumanPBMCs_RevE.pdf). Samples were thawed at 37°C for 3 min. This was followed by stepwise dilution (5x 1:1) with dropwise addition of complete growth medium (10% FBS + 90% RPMI). The sample was then filtered using a 50 µm strainer and centrifuged at 300g for 5 min at RT.
Supernatant was removed to the last millilitre and the cell pellet was resuspended in it by using a wide bore pipet. After slowly adding another 9 ml of complete growth medium, the sample was split into two. A further centrifugation step at 300 rcf for 5 min at 4°C was performed.
One half of the sample was used for further processing for scRNA-seq analysis.
The other half of the sample was used to prepare the FACS analysis. The sample was incubated with 200µl Fc block (1:50) at 4°C for 10 min. Staining of the cells was done by a 20 min incubation with an antibody mastermix (1:400). After centrifugation at 300g for 7 min at 4°C, the cell pellet was resuspended in 300µl FACS buffer. The dead cells were stained immediately before flow cytometry.

In vitro monocyte phenotyping
Human monocytes were isolated from healthy donors as described above. 1x105 monocytes were seeded in a 96 well plate and co-incubated with plasma from patients with CCS or sterile ACS (at the different timepoints: TP1M-TP4M) (randomly selected) either in the presence of anti-hIL-6 (1µg/ml, invivogen, cat#mabg-hil6-3), isotype control (1µg/ml, invivogen, cat#mabg1-ctrlm) or in the absence of any additional treatment. After a 12 h incubation at 37°C and 5% CO2, the cells underwent a washing step and were then incubated with Fc-Block (1:100, BD Biosciences, cat#564220) for 10 min, followed by incubation with the respective primary antibodies (1:100, see antibody panel) for 20 min on ice in the dark. Following another wash step, flow cytometric analysis was conducted. Prior to FACS, live/dead staining was added.

In vitro T cell phenotyping
After isolation (as described above), PBMCs were stained with CFSE proliferation dye according to manufacturer's instruction (Invitrogen, cat#C34554). Upon completion of the staining, cells were resuspended in Roswell Park Memorial Institute (RPMI) 1640 medium supplemented with 10% fetal bovine serum (FBS), L-Glutamine, non-essential amino acids, sodium pyruvate and penicillin/streptomycin and platted in 96 well flat-bottom plates. Plasma derived from patients with CCS or different timepoints after sterile ACS (TP1M, TP2M, TP3M, TP4M) or CCS (TP0M) (randomly selected) was then added to each well at a plasma ratio of one to three. PBMC were incubated with the respective plasma for 96 h. T cell activation and phenotypical changes were then analyzed by flow cytometry and the average of at least 5 PBMC donors was calculated per incubated plasma.
Flow cytometry was performed as recently described113, 114. In brief, after 96 hours of incubation of isolated PBMC with plasma, cells were transferred into a 96-well round-bottom plate and centrifuged for 5 min at 400 g. Cells were then washed twice with ice cold PBS and incubated with human TrueStain FcX and fixable viability dye (eFluor 780, eBioscience™) for 15 min at room temperature. Next, cells were stained with perspective antibodies (see antibody panel) for 30 min at 4°. After 30 minutes, cells were again washed with ice cold PBS fixed with 1% paraformaldehyde solution. II. Flow cytometric data were analyzed using FlowJo V10.9.0 software.

In vitro Monocyte chemotaxis
Human monocytes were isolated as described above. Following counting, 2x105 monocytes were seeded into a 96 well plate and treated with either DMSO 1% (Sigma, cat#D2438) or SC144 (10µM, Selleckchem, cat#S7124) in monocyte medium for 2 h at 37°C, 5% CO2. For chemotaxis, after the incubation period, 1x105 monocytes were stained using CellTracker™ Red CMTPX (1:1000, Invitrogen, cat#C34552) for 10 min. Subsequently, monocytes were allowed to attach to the upper part of a 5 µm transwell insert (Sarstedt), and the transwell insert was transferred to an ultra-low attachment 24 well plate (Corning) containing monocyte medium (mentioned above) mixed with CCL2 (100 µg/ml, Bio-Techne, cat#279-MC/CF). After incubation for 2 h at 37°C and 5% CO2, cells were harvested from the lower chamber. To allow further detachment of cells, 5mM EDTA was added. Subsequently, the number of transmigrated cells was measured with flow cytometry and standardized to counting beads (CountBright™, Invitrogen).

In vitro monocyte ROS production
Following counting, 2x105 monocytes were seeded into a 96 well plate and treated with either DMSO 1% (Sigma, cat#D2438) or SC144 (10µM, Selleckchem, cat#S7124) in monocyte medium for 2 h, or were treated with CCS or sterile ACS plasma (randomly selected) at different timepoints for 12 h at 37°C, 5% CO2. Afterwards, monocytes were loaded with 2',7' DCFDA (5µM, Sigma) and incubated for 15 min at 37°C and 5% CO2. Subsequently, cells were exposed to PMA (200nM, Sigma) for one hour at 37°C and 5% CO2, followed by immediate flow cytometric analysis. SYTOX Red (1:1000, Invitrogen, cat#S34859) was added for live/dead staining.

Instrument

LSRFortessa Flow Cytometer (BD Biosciences)

Software

The flow cytometry data were analysed with FlowJo (BD, version: 10.8.1 or 10.9.0).

Flow Cytometry

Plots

Confirm that:

☒ The axis labels state the marker and fluorochrome used (e.g. CD4-FITC).

☒ The axis scales are clearly visible. Include numbers along axes only for bottom left plot of group (a 'group' is an analysis of identical markers).

☒ All plots are contour plots with outliers or pseudocolor plots.

☒ A numerical value for number of cells or percentage (with statistics) is provided.

Cell population abundance	Gating strategies of the coronary syndrome PBMC FACS data have been included in the manuscript
Gating strategy	Gating strategies of the coronary syndrome PBMC FACS data: Singlets were gated by SSC-W vs. SSC-H, live cells were gated by dead-cell negative gating, subsequently all CD45+ cells were selected, T-cells and B-cells were selected by CD3+, CD19- cells and CD3-, CD19+ cells respectively, T-cells were further subdifferentiated into CD4+ and CD8+ T-cells, non-T- non-B-cells were further subdifferentiated into CD14+ monocytes (which were further differentiated into CD14high, CD16 low monocytes; CD14 int CD16int monocytes and CD14 int, CD16 high monocytes, non monocytes were differentiated into CD56 + CD16+ NK cells, that were further differentiated into CD56 bright and CD56 dim cells based on CD56 expression and CD16 expression (since CD56 dim cells were CD16 high), non-NK, non-monocyte, non-T-, non-B-cells were gated through HLA-DR expression and HLA-DR+ cells were further subclustered to CD123+ pDCs and CD123- MDCs.

☒ Tick this box to confirm that a figure exemplifying the gating strategy is provided in the Supplementary Information.
